# Using a Multiplex Nucleic Acid *in situ* Hybridization Technique to Determine HCN4 mRNA Expression in the Adult Rodent Brain

**DOI:** 10.3389/fnmol.2019.00211

**Published:** 2019-08-28

**Authors:** Julia Oyrer, Lauren E. Bleakley, Kay L. Richards, Snezana Maljevic, A. Marie Phillips, Steven Petrou, Cameron J. Nowell, Christopher A. Reid

**Affiliations:** ^1^Florey Institute of Neuroscience and Mental Health, The University of Melbourne, Melbourne, VIC, Australia; ^2^School of Biosciences, The University of Melbourne, Parkville, VIC, Australia; ^3^Drug Discovery Biology, Monash Institute of Pharmaceutical Sciences, Monash University, Parkville, VIC, Australia

**Keywords:** HCN4 channels, HCN1 channel, HCN2, multiplex nucleic acid *in situ* hybridization, Ih, quantifcation workflow

## Abstract

Hyperpolarization-activated cyclic nucleotide-gated (HCN) channels carry a non-selective cationic conductance, I_*h*_, which is important for modulating neuron excitability. Four genes (*HCN1-4*) encode HCN channels, with each gene having distinct expression and biophysical profiles. Here we use multiplex nucleic acid *in situ* hybridization to determine HCN4 mRNA expression within the adult mouse brain. We take advantage of this approach to detect HCN4 mRNA simultaneously with either HCN1 or HCN2 mRNA and markers of excitatory (VGlut-positive) and inhibitory (VGat-positive) neurons, which was not previously reported. We have developed a Fiji-based analysis code that enables quantification of mRNA expression within identified cell bodies. The highest HCN4 mRNA expression was found in the habenula (medial and lateral) and the thalamus. HCN4 mRNA was particularly high in the medial habenula with essentially no co-expression of HCN1 or HCN2 mRNA. An absence of I_*h*_-mediated “sag” in neurons recorded from the medial habenula of knockout mice confirmed that HCN4 channels are the predominant subtype in this region. Analysis in the thalamus revealed HCN4 mRNA in VGlut2-positive excitatory neurons that was always co-expressed with HCN2 mRNA. In contrast, HCN4 mRNA was undetectable in the nucleus reticularis. HCN4 mRNA expression was high in a subset of VGat-positive cells in the globus pallidus external. The majority of these neurons co-expressed HCN2 mRNA while a smaller subset also co-expressed HCN1 mRNA. In the striatum, a small subset of large cells which are likely to be giant cholinergic interneurons co-expressed high levels of HCN4 and HCN2 mRNA. The amygdala, cortex and hippocampus expressed low levels of HCN4 mRNA. This study highlights the heterogeneity of HCN4 mRNA expression in the brain and provides a morphological framework on which to better investigate the functional roles of HCN4 channels.

## Introduction

Four genes (*HCN1-4*) encode HCN channels that generate a non-selective cation conductance, I_*h*_ (Biel et al., 2009; He et al., 2014). I_*h*_ influences a number of fundamental functions including the determination of the resting membrane potential of a neuron, the integration of dendritic synaptic input, the control of synaptic transmission, and the modulation of neuronal firing properties (Biel et al., 2009; He et al., 2014). HCN channels have also been implicated in a variety of pathophysiological states including epilepsy and pain (Reid et al., 2012; DiFrancesco and DiFrancesco, 2015; Tsantoulas et al., 2016). HCN channels exist as both homo- and heterotetramers. The four HCN homomeric channel subtypes have distinct biophysical properties [as reviewed in Biel et al. (2009), Sartiani et al. (2017)]. For example, the activation voltage of HCN1 channels is more depolarized compared to that of HCN2 and HCN4 channels. Activation kinetics also differ, with the rate of channel activation being fastest for HCN1 and slowest for HCN4. Furthermore, HCN channel subtypes are modulated differently, with HCN2 and HCN4 channels being highly sensitive to cAMP concentrations, while HCN1 and HCN3 are less so. Heteromeric HCN channels tend to have hybrid properties extending the biophysical functional diversity of this channel class. These biophysical differences are tuned to specific functions in the brain (Lüthi and McCormick, 1999; Biel et al., 2009). Complementing this functional diversity is the distinct regional expression of the various HCN channel subunits throughout the brain (Santoro et al., 2000; Notomi and Shigemoto, 2004).

Despite the longstanding knowledge that HCN4 channels are expressed in the central nervous system, their role has been relatively understudied compared to HCN1 and HCN2 channels. Recent physiological studies in conditional HCN4 knockout mice reveal a role for these channels in controlling rhythmic intra-thalamic oscillations, presumably through driving intrinsic burst-firing in thalamocortical neurons (Zobeiri et al., 2019). From a pathophysiological perspective, there is some evidence that functional genetic variants are associated with epilepsy (Becker et al., 2017; Campostrini et al., 2018; DiFrancesco et al., 2019). HCN4 mRNA levels are also increased in the pilocarpine rodent model of temporal lobe epilepsy (Surges et al., 2012), while in a cortical stroke model in which seizures develop, there is a switch from HCN2 to HCN4 channel expression in thalamocortical neurons (Paz et al., 2013). A clear anatomical map of HCN4 channel expression is key to better understanding the physiological and pathophysiological roles of these channels. Previous studies have used traditional *in situ* hybridization and immunohistochemical methods to map HCN4 mRNA and HCN4 protein expression in the brain (Moosmang et al., 1999; Santoro et al., 2000; Hughes et al., 2013; Günther et al., 2019; Zobeiri et al., 2019). Here we take advantage of a multiplex *in situ* hybridization technique to map the expression of HCN4 mRNA in VGlut1, VGlut2, and VGat-positive neurons in different anatomical loci of the rodent brain. We also map the co-expression of HCN4 mRNA with both HCN1 and HCN2 mRNA.

## Materials and Methods

### Animals

Experiments were carried out as approved by The Florey Institute of Neuroscience and Mental Health Animal Ethics Committee and in accordance with the guidelines of the NHMRC of Australia Code of Practice for the Care and Use of Animals for Experimental Purposes. Male C57BL/6J mice aged P40 – P42 were used for RNAscope^®^ experiments (three mice for HCN4/HCN2 combination and three mice for HCN4/HCN1 combination). Tissue for Western blots was taken from one Nestin-Cre male and one Nestin-Cre x HCN4-floxed male, aged P31 and P37, respectively. For electrophysiological studies, four Nestin-HCN4 knockout mice (3 male and 1 female) aged P36 – P45 were used, together with four (3 male and 1 female) Nestin-Cre mice aged P29 - P35 for controls.

### Western Blot

Mice were deeply anesthetized using isoflurane, decapitated, dissected to isolate thalamus-enriched tissue, and the tissues were stored at −80°C. The isolated tissues were lysed in RIPA buffer (10 mM Tris pH 8.0, 1% Triton X100, 0.1% sodium deoxycholate, 1% SDS 140 mM sodium chloride) and made to a final protein concentration of 5 μg/μl protein, 2 M Urea, and 1xSDS reducing loading buffer (Laemmli, 1970). Known protein concentrations (100 μg) of control and experimental samples were loaded on a gel (7% poly-acrylamide SDS PAGE), transferred to nitrocellulose, and satisfactory transfer of protein by Western blotting monitored by Ponceau S (Sigma-Aldrich; Cat No. P3504) staining. Filters were blocked in 0.5% skim milk 1xTBS 1.0% Igepal (Sigma-Aldrich; Cat No. I3021) for 1 h, incubated overnight at 4°C with primary antibodies [mouse anti-HCN4 (Neuromab; Cat No. 73-150) 1:500], washed, and incubated for 1 h at room temperature with secondary antibodies [goat anti-mouse poly HRP (Invitrogen, Cat No. 32230) 1:15,000]. The protein signal was visualized with Clarity Western ECL Substrate (BioRad; Cat No. 170-5061) and the signal captured by a BioRad ChemiDoc MP System and quantitated with ImageJ.

### Electrophysiology

Mice were deeply anesthetized using isoflurane and decapitated. Brains were rapidly removed and mounted in a slice chamber containing chilled cutting solution (125 mM choline chloride, 20 mM D-Glucose, 0.4 mM CaCl_2_^⋅^2H_2_O, 6 mM MgCl_2_^⋅^6H_2_O, 2.5 mM KCl, 1.25 mM NaH_2_PO_4_ and 26 mM NaHCO_3_). 300 μm thick coronal slices were cut using a vibratome (Leica VT1200 S). Slices were held in a holding chamber with artificial cerebrospinal fluid (125 mM NaCl, 10 mM D-Glucose, 2 mM CaCl_2_^⋅^2H_2_O, 2 mM MgCl_2_^⋅^6H_2_O, 2.5 mM KCl, 1.25 mM NaH_2_PO_4_, and 26 mM NaHCO_3_) and bubbled continuously with carbogen gas at 32°C for at least 15 min and then room temperature for at least 30 min before recording. Whole-cell recordings from the medial habenula were made using borosilicate glass electrodes (3–6 MΩ) filled with an internal solution comprising 125 mM K-gluconate, 5 mM KCl, 2 mM MgCl_2_^⋅^6H_2_O, 10 mM HEPES, 4 mM ATP-Mg, 0.3 mM GTP-Na, 10 mM phosphocreatine, 0.1 mM EGTA, and 0.2% biocytin; with a pH of 7.2 and osmolarity of 291 mOsm. Bridge balance and capacitance neutralization were applied. Neurons were maintained at a membrane potential of −50 mV with a 5 s −70 pA current injection used to elicit I_*h*_-mediated “sag.” The start-to-start interval was 6.5–7 s. A response over 5–10 sweeps was averaged. Sag was calculated as the difference between the most hyperpolarized point within the first second of the average trace and the final “steady state” of this trace.

### Multiplex *in situ* Hybridization RNAscope^®^

#### Tissue Preparation

Mice were deeply anesthetized using isoflurane and decapitated. Brains were rapidly removed and snap frozen in a liquid nitrogen vapor phase. Tissue was stored at −80°C until sectioned.

The fresh-frozen brain was embedded in optimum cutting temperature (OCT) embedding gel (Tissue-Tek^®^, Sakura Finetek, United States). Coronal brain slices were cut at 15 μm thickness using a cryostat (CM 1800, Leica Microsystems) and mounted on positively charged microscope slides. Coronal sections were cut according to the Paxinos Mouse Brain Atlas (approximately bregma −1.70 mm) (Paxinos and Franklin, 2001). Cryosections were kept at −20°C during sectioning and stored for up to 3 weeks at −80°C until further processed for RNAscope^®^ [advanced cell diagnostics (ACD), Newark, CA, United States].

#### RNAscope^®^ Fluorescent Multiplex Assay

RNA *in situ* hybridization was performed using RNAscope^®^ (ACD) (Wang et al., 2012). Standard mouse probes were used to examine the expression of HCN1 (ACD; Cat No. 423651-C2), HCN2 (ACD; Cat No. 427001-C2), and HCN4 (ACD; Cat No. 421271) mRNA. Probes to VGlut1 (Slc17a7-C2) (ACD; Cat No. 416631-C2), VGlut2 (Slc17a6-C3) (ACD; Cat No. 319171-C3), and VGat (Slc32a1-C3) (ACD; Cat No. 319191-C3). mRNA were used to mark excitatory and inhibitory neurons, respectively. DAPI (ACD; Cat No. 320850) was employed as a cell nucleus marker. Positive (mixture of three probes including Polr2a on C1, PPIB on C2, UBC on C3; ACD; Cat No. 320881) and negative (DapB; ACD; Cat No. 320871) control probes were used to test optimal permeabilization and probe RNA quality.

Tissue was processed according to the protocol set out in the user manual. Briefly, sections were fixed in 4% paraformaldehyde (PFA) for 15 min at 4°C and dehydrated for 5 min each at room temperature and with increasing concentrations of ethanol (50, 70, 100, and 100%) diluted in (RNase-free) diH_2_O. Sections were air-dried for 5 min at room temperature and a hydrophobic barrier was drawn around each section using an ImmEdge Hydrophobic Barrier Pen (Vector Labs; Cat No. 310018). Sections were then incubated with protease pretreatment 4 (ACD; Cat No. 322340) for 30 min at room temperature and then washed twice in PBS (1x; RNase-free) before being incubated for 2 h at 40°C with three Mus musculus (Mm) multiplexed probe combinations: (a) Mm-HCN4 (channel 1), Mm-HCN1 (channel 2), and Mm-Slc32a1 (channel 3); (b) Mm-HCN4 (channel 1), Mm-HCN2 (channel 2), and Mm-Slc17a6 (channel 3); and (c) Mm-HCN4 (channel 1), Mm-Slc17a7 (channel 2), and Mm-Slc32a1 (channel 3). For each combination, sections from a minimum of three animals were processed in independent experiments. After hybridization, sections were washed twice in wash buffer (ACD; Cat No. 31009). Signals were amplified according to the protocol using four consecutive amplification steps with the fluorescent multiplex reagent kit (ACD; Cat No. 320850). For amplification step 4, the fluorescent label combination AltB was used. Sections were then washed twice using wash buffer, after which the sections were stained with DAPI for 30 s, covered with ProLong Diamond antifade reagent (Thermo Scientific Fisher; Cat No. P36962) and cover slipped. Company supplied positive and negative controls were both used to confirm specificity of staining.

#### Imaging

**FIGURE 1:**
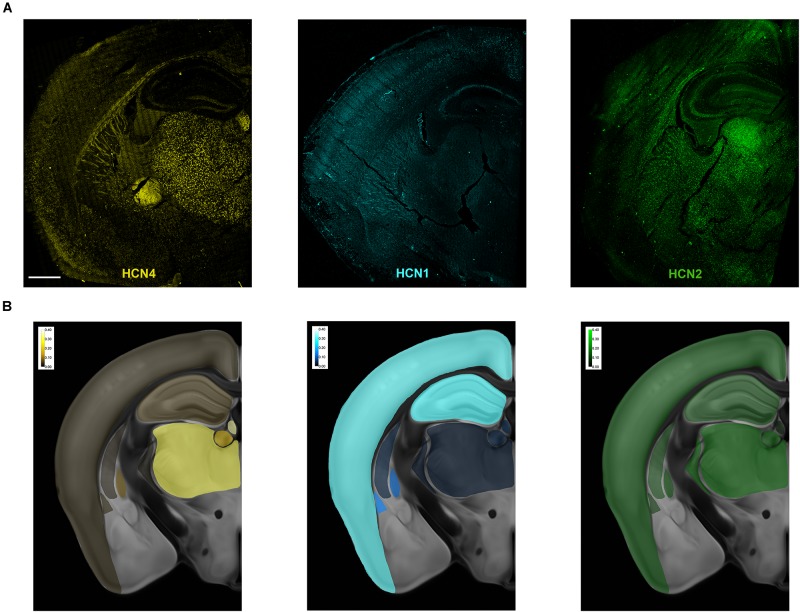
Overview of HCN mRNA expression in the mouse brain. **(A)** Representative maximum intensity projection of a coronal brain slice showing HCN4, HCN2, and HCN1 mRNA expression. In these overview images, brightness, contrast, and γ (HCN4 0.6, HCN1 0.5, and HCN2 0.5) were adjusted purely for illustration purposes. The bright uniform staining below the thalamus in the HCN4 image is an optical aberration and was not analyzed. **(B)** Heatmaps of relative levels of mRNA for each HCN mRNA expressed as a% of total number of cells. Scale bar = 700 μm.

The sections were imaged on an 8 kHz resonant scanning confocal microscope using a pixel dwell time of 72 ns (Leica TCS SP8, Leica GmbH) with a U Plan Apo 40× oil immersion objective with a numerical aperture of 1.3. Complete coronal brain slices were captured using the tile scanning module and Z series were collected to compensate for focal drift. Images were acquired sequentially. Excitation (ex) and emission (em) spectra were set as follows: DAPI (ex 405 nm, em 411–490 nm) on Photomultiplier Tube (PMT); Alexa 488 (ex 488 nm, ex 500–540 nm) on Hybrid Detector (HyD); Atto 550 (ex 561 nm, em 565–620 nm) on HyD; and Alexa 647 (ex 633 nm, em 645–705 nm) on PMT. For quantification analysis, images were taken as single planes. To generate single images in Figure 1, maximum intensity projections of Z series were created. Brightness and contrast were adjusted just above saturation for illustration purposes.

#### RNAscope^®^ Quantification

For each coronal slice, areas from a single opitcal plane were extracted according to “The Mouse Brain in stereotaxic coordinates” and “The Allen Mouse Brain Reference Atlas” (Paxinos and Franklin, 2001; Lein et al., 2007). The extracted areas were as follows: thalamus [without habenula and thalamic reticular nucleus (NRT)], habenula, NRT, striatum, globus pallidus external (GPe), hippocampus, somatosensory cortex, and retrosplenial cortex. Extracted areas were analyzed with a custom-built Fiji macro code (Schindelin et al., 2012). In brief, the macro code performed the following steps: nuclei were extracted using standard watershed segmentation. RNAscope channels were filtered for noise using a Gaussian (sigma = 1) filter. RNAscope positive foci were extracted using maximal point extraction with the user-defined tolerance values appropriate for each sample. Nuclei masks were dilated by three pixels to cover more of the “cell” area. The resulting nuclei areas were then used to count the number of RNAscope foci in each given cell. A cell was deemed positive if it contained three or more RNAscope foci. Our quantitative analysis method was not applied to brain regions where HCN4 mRNA expression was less than 4 dots per DAPI-defined cell or if cell bodies could not be separated due to their high density. The full macro code is available for download from https://doi.org/10.26180/5cde05da74ce0. For each extracted area a minimum of three coronal slices from three different animals was analyzed.

#### Statistics

All RNAscope data were directly extracted from the macro code and are presented as individual values and mean values. Error bars represent the standard error of the mean (SEM). For electrophysiological data, a Shapiro-Wilk test was initially applied to test for normality, and returned a value of >0.05 in all groups. Statistical significance was then determined using an unpaired two-tailed Student’s *t* test (GraphPad Prism version 8.1, GraphPad Software).

## Results

### Overview of HCN4 mRNA Expression in the Mouse Brain

Multiplex *in situ* hybridization was used to map the expression of HCN4, HCN1 and HCN2 mRNA in a single brain plane (approximately bregma −1.70 mm, Figure 1A). Maximum projection images of each subunit are presented in Figure 1A. The uniform staining seen below the thalamus in the HCN4 image is an optical aberration. Similarly, fluorescence observed in the fiber tracts in this image is uniform and due to auto-fluorescence. These areas were not quantified. Heatmaps showing the relative expression of each subunit across the brain slice are presented in Figure 1B. Expression of HCN4 mRNA was highest in thalamus and medial habenula but was also found in the globus pallidus external (GPe), striatum, hippocampus and cortex. We used a multiplex *in situ* hybridization technique to investigate the pattern of co-expression of HCN4 mRNA with HCN1 and HCN2 mRNA. VGlut1, VGlut2, and VGat were used to establish neuron type.

A Fiji-based code was developed to quantify mRNA expression^1^. The cell body was defined by the nuclear marker DAPI with cell volume estimated by expanding the boundaries by 3 pixels. We use this quantitative approach to describe HCN4 mRNA expression in brain regions where we were able to separate individual DAPI marked cells and where mRNA clustered within the cell body. We structure the results by first focusing on brain regions that express the highest HCN4 mRNA levels and progressively describe brain regions with lower expression.

### HCN4 mRNA Is Abundant in the Habenula

**FIGURE 2:**
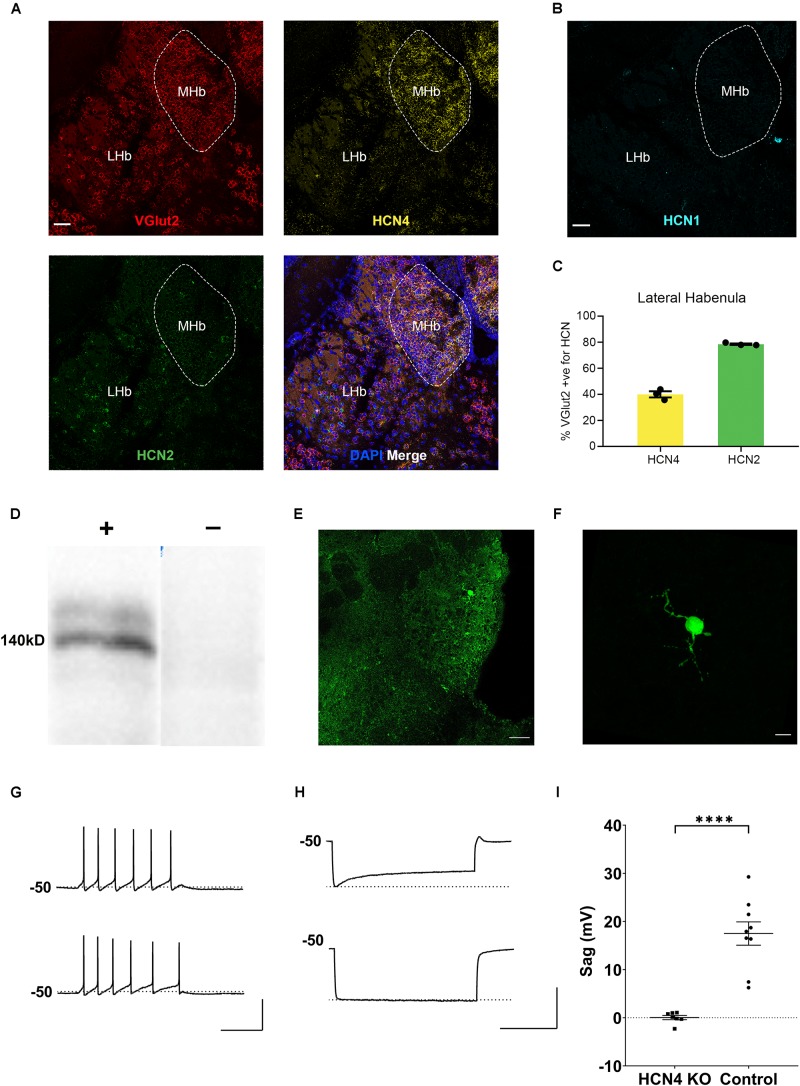
HCN4 channels support I_*h*_ in the medial habenula. **(A)** Representative confocal images (single optical slice) of the lateral and medial habenula (LHb, MHb) showing VGlut2, HCN4, and HCN2 mRNA expression presented individually and as a composite with DAPI staining. Scale bar = 50 μm. **(B)** Representative confocal images (single optical slice) of LHb and MHb showing HCN1 mRNA expression from a different slice. Scale bar = 50 μm. **(C)** Percentage of VGlut2-positive neurons in the LHb expressing HCN4 or HCN2 mRNA. **(D)** Western blot analysis of a Nestin-HCN4 knockout mouse (–) and Nestin-Cre control (**+)**. **(E)** Biocytin-filled neuron identifying a recorded neuron in the medial habenula. Scale bar = 50 μm. **(F)** Maximum intensity projection of the identified neuron. Scale bar = 10 μm. **(G)** Example traces showing action potential firing at rheobase in a medial habenula neuron from a Nestin-Cre control mouse (upper) and from a Nestin-HCN4 knockout mouse (lower). Scale bar = 200 ms (*x* axis), 50 mV (*y* axis). **(H)** Example traces of “sag” on the voltage recording (average of 5–10 sweeps) from a Nestin-Cre mouse (upper) and from a Nestin-HCN4 knockout mouse (lower). Scale bar = 2 s (x axis), 50 mV (y axis). **(I)** Scatter plot comparing “sag” in medial habenula neurons from Nestin-HCN4 knockout mice (7 cells) and Nestin-Cre mice (9 cells). ^∗∗∗∗^*p* < 0.0001.

HCN4 mRNA is very highly expressed in the habenula (Figure 2A; Moosmang et al., 1999; Santoro et al., 2000). The habenula can be broadly divided into the lateral and medial habenula, with each playing a distinct physiological role (Mizumori and Baker, 2017; Lee et al., 2019). The medial habenula expressed high levels of HCN4 mRNA (Figure 2A). However, cells are too densely packed in this region to allow quantitative expression analysis. The HCN4 mRNA signal was also present in the lateral habenula in which quantitative analysis revealed HCN4 mRNA in 19 ± 2% (*n* = 7 slices) of DAPI-identified cells. In the same region, HCN2 mRNA was expressed in 59 ± 3% (*n* = 3 slices), while HCN1 expression was only seen in 0.9 ± 0.4% (*n* = 4 slices) of cells (Figure 2B). The vast majority of HCN4 mRNA positive-cells (89 ± 1%, *n* = 3 slices) co-expressed VGlut2 (Figure 2C), a marker of excitatory neurons. Furthermore 90 ± 2% (*n* = 3 slices) of HCN4 mRNA-positive cells also expressed HCN2 mRNA (Figure 2C). This indicates that HCN4 channels potentially co-express with HCN2 channels in excitatory neurons in the lateral habenula.

In contrast to HCN4, HCN1, and HCN2 mRNA expression was essentially undetectable in the medial habenula (Figures 2A,B). This implies that HCN4 channels are the major and potentially the sole carrier of I_*h*_ in this region. To test this hypothesis, whole-cell electrophysiological recordings were made from neurons identified in the medial habenula in Nestin-HCN4 knockout mice (Figures 2D,E). Recorded neurons had a small dendritic arbor (Figure 2F) with depolarizing current injections mostly generating tonic trains of action potential firing (Figure 2G). In all Nestin-Cre control neurons, hyperpolarizing current injections generated an I_*h*_-mediated “sag” on the recorded voltage (Figures 2H,I). In brain-specific HCN4 knockout mice, I_*h*_-mediated “sag” was absent, strongly supporting the role of HCN4 channels as the exclusive mediator of this current in the medial habenula (Figures 2H,I).

### HCN4 mRNA Is Abundant in the Thalamus

**FIGURE 3:**
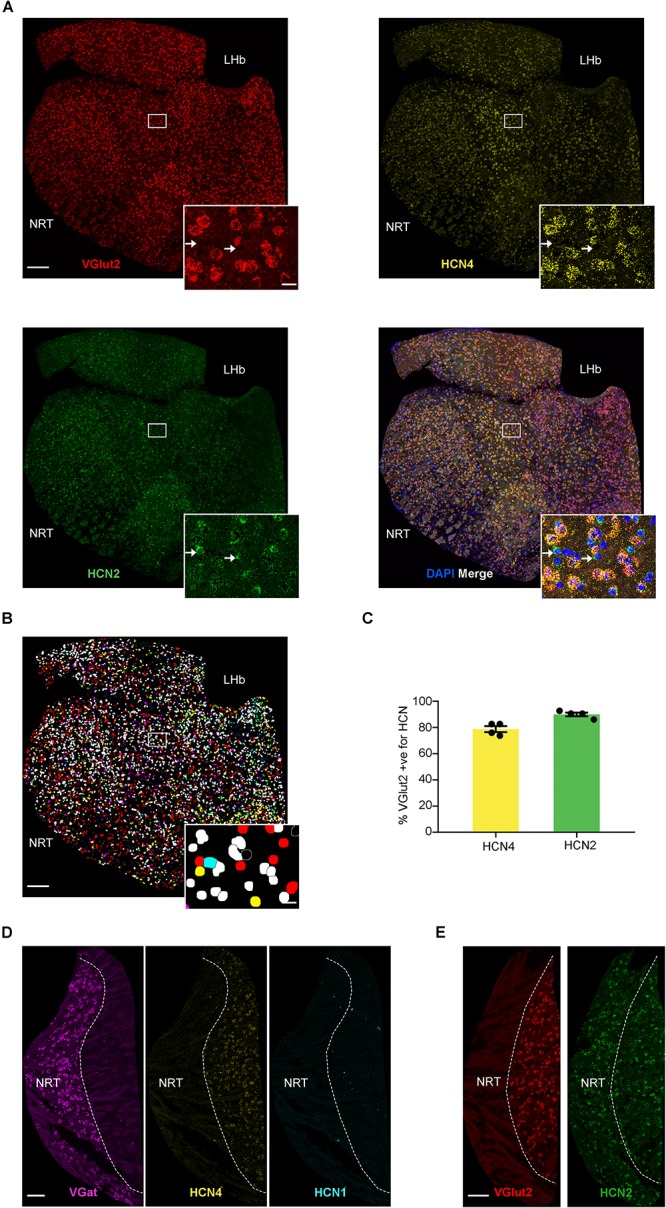
High HCN4 mRNA expression in the thalamus. **(A)** Representative confocal images (single optical slice) of the thalamus showing VGlut2, HCN4, and HCN2 mRNA expression presented individually and as a composite with DAPI staining. For analysis, thalamus regions (excluding NRT and habenula) were cut out and areas other than the thalamus were masked as presented here. Scale bar = 200 μm. Inset scale bar = 20 μm. **(B)** Classification of thalamic cell types based on combination of mRNA expressed. Blue, HCN4; red, HCN2; green, VGlut2; yellow, co-expression of HCN2 and VGlut2; cyan, co-expression of VGlut2 and HCN4; magenta, co-expression of HCN2 and HCN4; white, triple expression. **(C)** Percentage of VGlut2-positive neurons that express HCN4 or HCN2 mRNA. **(D)** Representative confocal images (single optical slice) of the NRT showing VGat mRNA, HCN4 mRNA, and HCN1 mRNA. Scale bar = 100 μm. **(E)** Representative confocal images (single optical slice) of NRT showing VGlut2 and HCN2 mRNA. Scale bar = 100 μm.

As shown in previous studies, HCN4 mRNA was also highly expressed in the thalamus (Figure 3; Moosmang et al., 1999; Santoro et al., 2000). We completed analysis on thalamic sub-nuclei combined (excluding NRT and habenula) and measured HCN4 mRNA in 34 ± 3% (*n* = 8 slices) of cells. Using VGlut2 as a marker of excitatory thalamocortical neurons (Varoqui et al., 2002; Fremeau et al., 2004), 43 ± 5% (*n* = 4 slices) of cells were found positive for VGlut2 mRNA in the thalamus. HCN2 mRNA was seen in 67 ± 3% (*n* = 4 slices), with HCN1 mRNA essentially undetectable (0.6 ± 0.2%, *n* = 4 slices). The vast majority of VGlut2 positive cells expressed HCN4 and HCN2 mRNA (Figures 3A,B). HCN4 mRNA was only detected in VGlut2-positive neurons (90 ± 2%, *n* = 4 slices) and was always co-expressed with HCN2 mRNA (95 ± 1%, *n* = 4 slices) (Figure 3C). In contrast, only 58 ± 5% (*n* = 4 slices) of cells positive for HCN2 mRNA co-expressed VGlut2. This indicates that HCN2 is also expressed in cell types that are VGlut2- and HCN4-negative (see arrows Figure 3A).

### HCN4 mRNA Is Very Low in the Thalamic Reticular Nucleus

The presence of VGat mRNA and absence of VGlut1 and VGlut2 mRNA is consistent with the predominance of GABAergic neurons in the NRT (Figures 3D,E; Houser et al., 1980; de Biasi et al., 1986; Huguenard and McCormick, 1992). Only 1 ± 0.3% (*n* = 7 slices) of cells were positive for HCN4 mRNA, suggesting very low expression in the NRT. Similarly, HCN1 expression was low, with only 0.5 ± 0.3% (*n* = 4 slices) cells being positive (Figure 3D). In contrast, 61 ± 7% of cells (*n* = 3 slices) were positive for HCN2 mRNA, indicating that this is the predominant HCN channel subtype expressed in the NRT (Figure 3E).

### HCN4 mRNA Is Low in the Striatum but High in Putative Giant Cholinergic Interneurons

**FIGURE 4:**
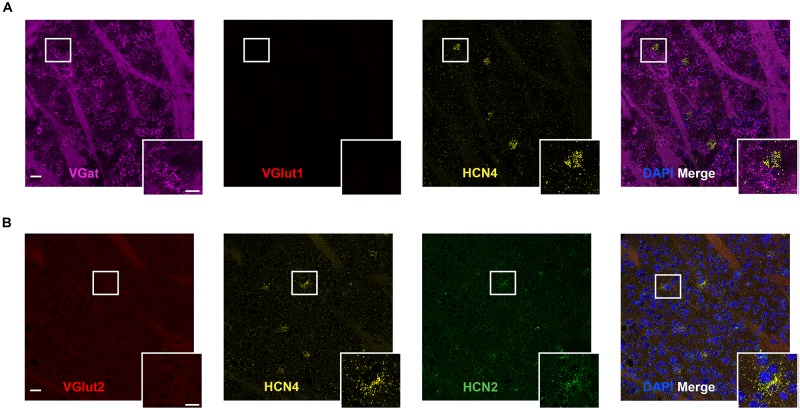
High HCN4 mRNA expression in giant cholinergic neurons in the striatum. **(A)** Representative confocal images (single optical slice) in the striatum showing VGat, VGlut1, and HCN4 mRNA presented individually and as a composite with DAPI staining. Co-expression of magenta and yellow = white. Scale bar = 30 μm. Inset scale bar = 15 μm. **(B)** Representative confocal images (single optical slice) in the striatum showing VGlut2, HCN4, and HCN2 mRNA presented individually and as a composite with DAPI staining. Scale bar = 30 μm. Inset scale bar = 15 μm.

The striatum expressed HCN4 mRNA at very low levels (Figure 4). Only 2 ± 1% (*n* = 4 slices) of cells were positive for HCN4 mRNA, but in these cells, expression was very high (Figure 4). Further inspection revealed that HCN4 mRNA was only labeled in a small subset of very large cells (Figure 4). These cells did not express VGlut1, VGlut2 or VGat mRNA (Figure 4), suggesting that they were cholinergic interneurons, which comprise about 1–2% of striatal cells (Bolam et al., 1984; Contant et al., 1996; Lim et al., 2014). In the striatum, HCN2 mRNA was more widely expressed (40 ± 5% of cells, *n* = 4 slices), while HCN1 expression was very low (0.8 ± 0.4%, *n* = 3 slices). HCN4 mRNA-positive cells mostly co-expressed HCN2 mRNA (84 ± 10% of cells, *n* = 4 slices), while co-expression of HCN1 mRNA was essentially non-existent (2 ± 2% of cells, *n* = 3 slices).

### HCN4 mRNA Is Low in the Globus Pallidus External (GPe) and Expressed Almost Exclusively in VGat-Positive Cells

**FIGURE 5:**
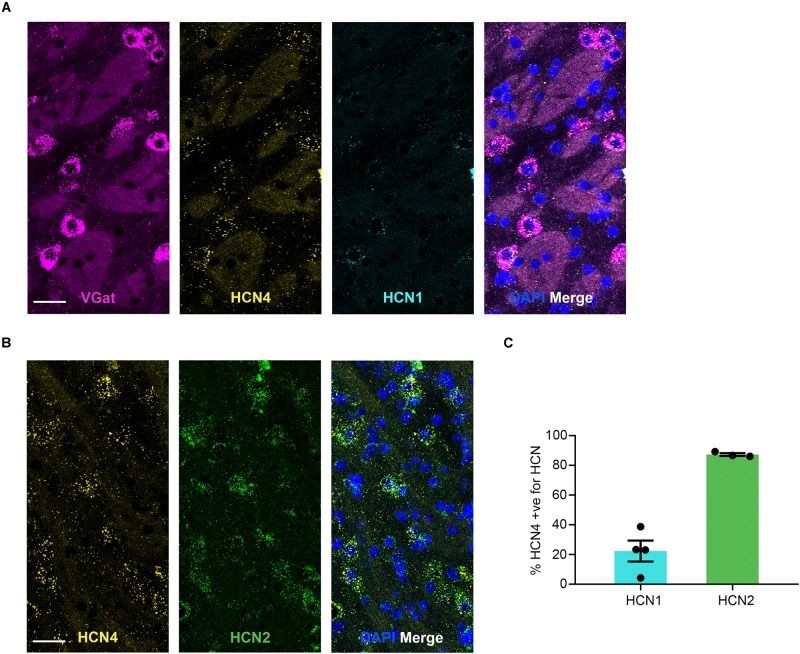
HCN4 mRNA expression in GABAergic neurons in the globus pallidus external. **(A)** Representative confocal images (single optical slice) in the globus pallidus external showing VGat, HCN4, and HCN1 mRNA presented individually and as a composite with DAPI staining. Co-expression of magenta and yellow = white. Scale bar = 30 μm. **(B)** Representative confocal images (single optical slice) in the globus pallidus external showing HCN4 and HCN2 mRNA presented individually and as a composite with DAPI staining. Scale bar = 30 μm. **(C)** Percentage of HCN4 positive neurons that are expressing HCN1 or HCN2 mRNA.

HCN4 mRNA was expressed at low levels in the GPe (Figure 5) with 8 ± 2% (*n* = 7 slices) of cells positive. 78 ± 5% (*n* = 4 slices) of HCN4 mRNA-positive cells co-expressed VGat mRNA, indicating that these channels are predominantly expressed in GABAergic neurons in this region. However, 29 ± 3% (*n* = 4 slices) of VGat mRNA-positive cells co-expressed HCN4 mRNA, suggesting that HCN4 mRNA is only present in a subset of GABAergic neurons. In the GPe, HCN1 mRNA was also expressed at low levels (3 ± 0.8% of cells, *n* = 4 slices), while HCN2 mRNA was expressed more widely (49 ± 6% of cells, *n* = 3 slices). The majority of HCN4 mRNA-positive cells also expressed HCN2 mRNA, with a smaller subset co-expressing HCN1 mRNA (Figure 5).

### Low HCN4 mRNA Expression in the Hippocampus

**FIGURE 6:**
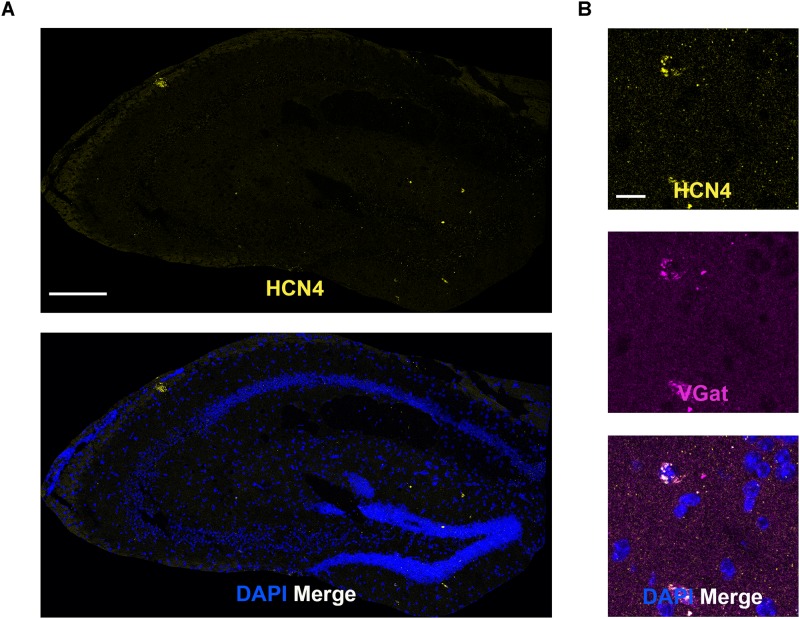
Low HCN4 mRNA expression in the hippocampus. **(A)** Representative confocal images (single optical slice) in the hippocampus showing HCN4 mRNA presented individually and as a composite with DAPI staining. Scale bar = 250 μm. **(B)** Representative confocal images (single optical slice) in the dentate gyrus showing HCN4 and VGat mRNA expression presented individually and as a composite with DAPI staining. Co-expression of magenta and yellow = white. Scale bar = 20 μm.

In general, HCN4 mRNA expression was very low and diffuse (not clustered) in the hippocampus, making quantitative analysis unreliable. We specifically looked at co-expression using the GABAergic marker, VGat, given that HCN4 channel expression has been reported in parvalbumin-positive neurons (Hughes et al., 2013; Seo et al., 2015). Consistent with those findings, HCN4 mRNA clustered in a subset of VGat-positive neurons (Figure 6). Quantitative analysis revealed that 15 ± 8% of VGat positive cells (*n* = 3) slices expressed HCN4 mRNA, although it should be noted that this is only a rough estimate given the low expression.

### HCN4 mRNA Expression in Other Brain Regions

**FIGURE 7:**
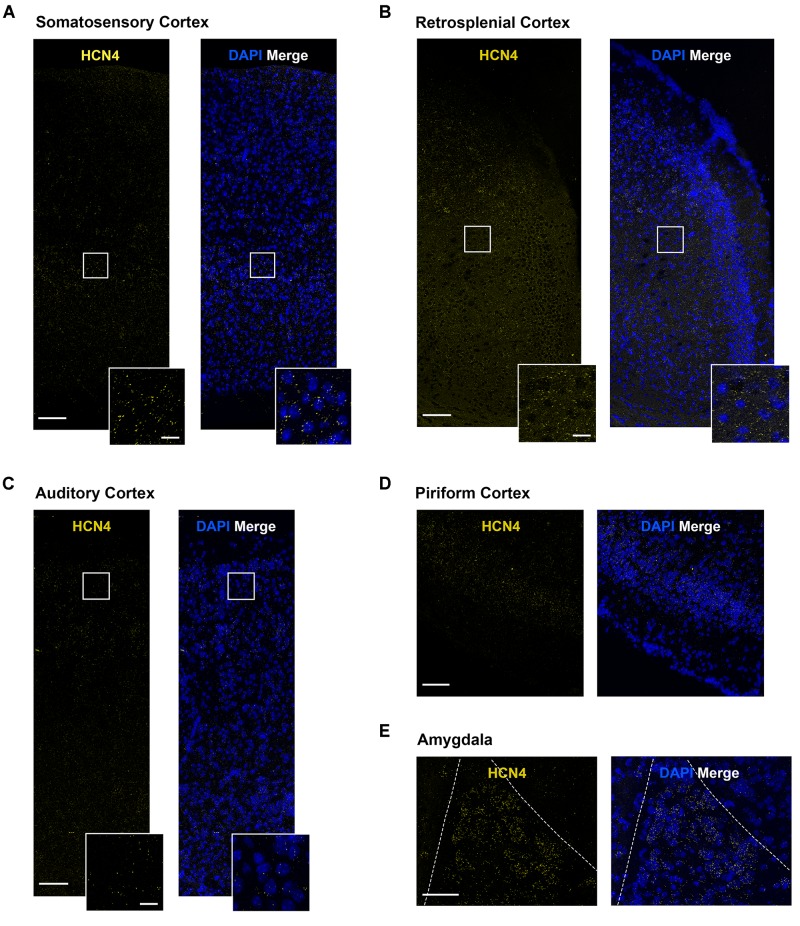
HCN4 mRNA expression in the cortex and the amygdala is low and diffuse. **(A)** Representative confocal images (single optical slice) in the somatosensory cortex showing HCN4 mRNA presented individually and as a composite with DAPI staining. Scale bar = 100 μm. Inset scale bar = 20 μm. **(B)** Representative confocal images (single optical slice) in the retrosplenial cortex showing HCN4 mRNA presented individually and as a composite with DAPI staining. Scale bar = 100 μm. Inset scale bar = 20 μm. **(C)** Representative confocal images (single optical slice) in the auditory cortex showing HCN4 mRNA presented individually and as a composite with DAPI staining. Scale bar = 100 μm. Inset scale bar = 20 μm. **(D)** Representative confocal images (single optical slice) in the piriform cortex showing HCN4 mRNA presented individually and as a composite with DAPI staining. Scale bar = 100 μm. **(E)** Representative confocal images (single optical slice) in the amygdala showing HCN4 mRNA presented individually and as a composite with DAPI staining. Scale bar = 80 μm.

HCN4 mRNA expression was above background in a number of other brain regions. However, in these regions the expression pattern was generally diffuse, making quantitative analysis unreliable. We have therefore only presented the HCN4 mRNA probe results in these regions without interpreting co-expression with other HCN channels or neuron markers. These regions include the somatosensory, auditory, piriform and retrosplenial cortices (Figures 7A–D), and the lateral amygdala (Figure 7E). Importantly, although quantification was not possible, our results show HCN4 mRNA expression above background and suggest that HCN4 channels have a functional role in these regions.

## Discussion

Multiplex nucleic acid *in situ* hybridization provides a powerful method to probe mRNA expression in tissue with improved specificity and sensitivity, as well as the ability to use multiple probes simultaneously (Wang et al., 2012). Here we investigated the expression of HCN4 mRNA in the adult rodent brain. HCN4 mRNA was found to be highest in the thalamus and habenula (medial and lateral). HCN4 mRNA expression was also high in giant cholinergic interneurons in the striatum and in a subset of VGat-positive interneurons in the GPe. The cortex, amygdala and hippocampus had lower, more diffuse expression patterns for HCN4 mRNA. These results generally recapitulate previous work using standard *in situ* techniques (Moosmang et al., 1999; Santoro et al., 2000). Importantly, we were able to extend these previous results by identifying the neuron types that expressed HCN4 mRNA using markers of excitatory and inhibitory neurons, and by establishing the co-expression pattern of HCN4 mRNA with other HCN channel subtypes in a subset of brain regions.

A workflow based on a Fiji macro enabled the quantification of mRNA expression within cell bodies identified by the nuclear marker, DAPI. This provides a powerful tool for understanding mRNA expression profiles. However, there are some important caveats to this approach. First, the method only works in regions of the brain in which cell bodies are separated sufficiently to identify single cells. In regions such as the medial habenula and piriform cortex, the high density of cell bodies precludes such analysis at the resolution imaged here. Second, the method can only provide an estimate of mRNA expression in the defined cell body. This cannot take into account mRNA in other compartments such as dendrites and axons. Similarly, mRNA in dendrites and axons that cross the cell body may “contaminate” the signal. This analytical method therefore works best for situations where high mRNA expression is clustered at the cell body and should be avoided when mRNA expression is diffuse, as seen in the cortex and hippocampus for HCN4 mRNA. Furthermore, although the multiplex *in situ* hybridization technique allows high resolution mapping of HCN mRNA, the method cannot report on protein levels or post-translational aspects of function. This is especially important given that HCN channels are subject to a number of different post-translational mechanisms in both physiological and pathological settings, resulting in biophysical and trafficking changes (Lewis et al., 2010). This said, functional analysis at the cellular level in the different brain regions is largely consistent with HCN4 mRNA expression patterns observed in our study.

HCN4 mRNA expression is high across both the lateral and medial habenula. I_*h*_ is a critical modulator of neuronal excitability in the lateral habenula (Yang et al., 2018). HCN4 mRNA was expressed in VGlut2-positive cells and mostly co-expressed with HCN2 mRNA in the lateral habenula. I_*h*_ has a hyperpolarized activation voltage of ∼−110 mV in this region, which is consistent with a heteromeric channel containing both HCN4 and HCN2 isoforms (Good et al., 2013). In the medial habenula, HCN4 mRNA expression is strikingly high, with expression around background levels for HCN1 and HCN2 mRNA (Moosmang et al., 1999; Santoro et al., 2000). At a cellular level, I_*h*_ underlies spontaneous tonic firing in cholinergic neurons suggesting HCN channels play an important role in defining excitability at least in a subset of medial habenula neurons (Görlich et al., 2013). Using whole-cell electrophysiological recordings from knockout mice we confirmed that HCN4 channels are the predominant subtype underlying I_*h*_ in these neurons.

The medial habenula can be subdivided, with distinct gene expression patterns reported for neurons in the superior-inferior, ventral-central, and lateral regions (Lecourtier and Kelly, 2007; Aizawa et al., 2012). Despite this, our results suggest a relatively uniform expression of HCN4 mRNA across the medial habenula. Further studies investigating the co-expression of HCN4 mRNA with markers of substance P, interleukin-18 and choline acetyltransferase are warranted.

HCN4 mRNA was also highly expressed in the thalamus, the main integrator and relay of sensory information to the cortex. The thalamus can be subdivided into a large number of nuclei, but given that HCN4 mRNA expression was uniform across the thalamic sub-nuclei, we combined them for analysis. Most VGlut2-positive excitatory neurons were positive for HCN4 mRNA and co-expressed HCN2 mRNA. Consistent with this, I_*h*_ is significantly reduced in thalamocortical relay neurons of both HCN2 and HCN4 knockout mice (Ludwig et al., 2003; Zobeiri et al., 2019). This strongly suggests that HCN4 and HCN2 subunits form heteromeric channels and that they are found in the majority of thalamocortical neurons. One interesting finding from our analysis is that HCN2 mRNA expressed alone in a subset of cells that were not positive for VGlut2 or HCN4 mRNA. The identity of these cells remains unknown.

HCN4 mRNA expression is high in a very select subset of cells in the striatum (Santoro et al., 2000). These cells are large and do not express VGlut1, VGlut2 or VGat mRNA, strongly suggesting that they are giant cholinergic interneurons. This needs confirmation using other markers including choline acetyltransferase and VGlut3 (Fremeau et al., 2002; Gras et al., 2002). At the cellular level, I_*h*_ is important for autonomous pacemaker and spiking activity in these interneurons (Wilson, 2005). HCN4 and HCN2 mRNA was co-expressed in these neurons, while HCN1 mRNA was essentially absent. This is broadly consistent with results reported using single-cell PCR (Zhao et al., 2016) although our results suggest approximately equal expression of HCN4 and HCN2 mRNA.

HCN4 mRNA expression was low and diffuse in the hippocampus. HCN4 channel expression has been reported in parvalbumin-positive neurons in the adult rodent hippocampus (Hughes et al., 2013; Seo et al., 2015). The expression of HCN4 mRNA in a subset of VGat-positive neurons is consistent with this. In a number of regions, such as the cortex and amygdala, HCN4 mRNA levels were above background but expression was not clustered. In these regions it is very difficult to make statements about co-expression and we only present HCN4 mRNA data. This does not mean that expression of the HCN4 channel is unimportant, or that it does not play critical functional roles in these brain areas. For example, despite the low expression of HCN4 mRNA in the cortex, immunohistochemical analysis reveals that HCN4 channel protein is robustly expressed, although at lower levels than those seen in the thalamus (Zobeiri et al., 2019). The precise functional role of HCN4 channels in the cortex and amygdala remains to be determined.

This study focused on high-resolution imaging of a single brain slice plane that encompassed regions known to express HCN4 channels, and established a robust workflow to measure mRNA expression using the multiplex *in situ* hybridization technique. HCN4 channels are known to be expressed in other central nervous system regions not investigated here, including the olfactory bulb, the cerebellum, and the spinal cord (Fried et al., 2010; Hughes et al., 2013; Nakashima et al., 2013; Zúñiga et al., 2016; Peng et al., 2017). Future studies using the multiplex *in situ* hybridization technique in these regions are warranted. Furthermore, we focused our efforts on co-expression of HCN4 mRNA with only broad markers of excitatory and inhibitory neurons. Additional markers of cell types, for example cholinergic or more specific GABAergic neuron markers as well as glia markers would refine the knowledge of expression of this channel. We also have not explored the expression of HCN3 mRNA. The functional importance of this channel is becoming evident (Kanyshkova et al., 2012; Stieglitz et al., 2017; Lainez et al., 2019) and the use of the multiplex *in situ* hybridization technique to map HCN3 mRNA expression is warranted. Finally, the multiplex *in situ* technique could be combined with immunohistochemical approaches to identify specific neuron compartments.

We have used the multiplex *in situ* hybridization technique to improve our knowledge about which neuron types express HCN4 mRNA. Furthermore, we have identified in several brain regions which other HCN channel subtypes co-express with HCN4 mRNA. These results provide a morphological framework on which to better investigate the functional roles of these important channels.

## Data Availability

All datasets generated for this study are included in the manuscript and/or the supplementary files.

## Ethics Statement

The animal study was reviewed and approved by The Florey Institute of Neuroscience and Mental Health Animal Ethics Committee and performed in accordance with the guidelines of the NHMRC of Australia Code of Practice for the Care and Use of Animals for Experimental Purposes.

## Author Contributions

JO, SP, and CR conceived the study. JO, LB, KR, and AP completed the experimental work. CN wrote the Fiji code for analysis of imaging data. JO and LB completed the analysis of data. All authors contributed to the writing of the manuscript.

## Conflict of Interest Statement

The authors declare that the research was conducted in the absence of any commercial or financial relationships that could be construed as a potential conflict of interest.

## Footnotes

1 https://doi.org/10.26180/5cde05da74ce0
